# X-linked hypophosphatemic osteomalacia with PHEX mutation presenting late in Pakistan

**DOI:** 10.1016/j.amsu.2021.01.067

**Published:** 2021-01-22

**Authors:** Nawazish Zehra, Lena Jafri, Salman Kirmani, Aysha Habib Khan

**Affiliations:** a Department of Pathology and Laboratory Medicine, Pakistan; b Department of Pediatric and Child Health Aga Khan University, Karachi, Pakistan

**Keywords:** Case report, Osteomalacia/rickets, PHEX mutation, Autosomal dominant

## Abstract

**Abstract.introduction.and.importance:**

Autosomal dominant hypophosphatemic rickets is the most common form of rare rickets, commonly manifests in children but sometimes the condition remains undiagnosed due to lack of knowledge &/or awareness of treating physicians or surgeons.

**Case presentation:**

We describe a case of 43 years old female with multiple fragility fractures since childhood, corrected surgically but never investigated. She had stunted growth, bowing deformities and loss of teeth.

**Clinical discussion:**

A detailed history and examination along with metabolic and genetic work up mounted the diagnosis of X linked hypophosphatemic osteomalacia. The pathophysiology involves the mutation or the loss of the phosphate regulating gene on PHEX, that causes reduced mineralization of bones and teeth.

**Conclusion:**

Diagnostic delay in this patient resulted in increased disabilities affecting her mobility and lif estyle.

## Highlights

• Rickets represents diseases of divergent causes, characterized by defect in matrix mineralization.
• Hypophosphatemic rickets is the most common form of autosomal dominant rickets.
• It involves the PHEX mutation.
• Awareness of these disorders among clinicians remains a diagnostic challenge because of their rarity and wide variety.

## 1 Introduction

Inherited metabolic diseases (IMDs) of bone are rare, with less than 1 per 200,000 incidence. Not only they have a heterogenous etiology, there is a wide variation in onset and disease severity [1]. Recently significant advances are made in understanding the underlying genetic and molecular pathways in the pathogenesis of rare bone diseases with new gene discovery and expansion of the classification system [1]. Almost 400 different forms of skeletal dysplasias have been described so far and the number continues to increase with advancement in molecular pathology. Worldwide the diagnostic capability and provision of accurate genetic counseling has been greatly improved. Increase comprehension of molecular pathways has also resulted in increase availability of therapeutic options for management of many disorders like osteogenesis imperfecta (OI), X-linked hypophosphatemia (XLH), primary hypoparathyroidism, osteopetrosis and hypophosphatasia. There is evolving interest in gene manipulation therapy too, to provide curative options for these often lethal disorders [2]. However, knowledge and awareness of these disorders among clinicians remains a diagnostic challenge because of their rarity and wide variety. But once diagnosed accurately patients may be treated appropriately, bone abnormalities prevented, emotional and financial devastation of the patients and their families may be compensated to some extent.

We describe a case presenting as adult with multiple bone deformities from childhood and treated surgically without diagnosis.This work is inline with the SCARE criteria 2020 [3,4]. (ref:scare criteria 2020)

### 1.1 Case presentation

A forty three years old, wheelchair-bound female presented to the metabolic bone clinic of Aga Khan University Hospital with short stature and grossly deformed bones of limbs and vertebrae. She achieved all milestones on time in childhood. She belonged to a financially sound family. The patient successfully passed her middle school and could not continue further studies because of her illness. There was no family history of having similar disorder and all of her siblings were in good state of health.

She developed low trauma fracture for the first time at eight years of age and was treated surgically. She had multiple low trauma fractures since childhood and surgical corrections with placement of rods were performed on multiple occasions. Her disability continued to increase with time and she was unable to walk and became wheelchair-bound. She lost all her teeth and was wearing dental prosthesis when examined in clinic. She also had a history of low trauma hip fracture one year back. She developed hypertension at the age of forty one years for which she was taking the combination of valsartan/hydrochlorothiazide 160/25mg.

On examination, the patient was short in height 4ft, with extreme bowing deformities involving leg, arms and back. She has light blue sclera, artificial teeth with clear oral hygiene. She had multiple scars of previous surgeries on arms and legs.

Results of the few biochemical investigations available with the patient included a normal complete blood count and lipid profile, serum calcium of 9.4 mg/dl, a serum 25 hydroxy vitamin D (25OHD) of 21.9 ng/ml, blood urea nitrogen and creatinine of 16 mg/dl and 0.8 mg/dl respectively. She was prescribed calcium supplements, and injection zoledronic acid. On her inquiry from physician, she was told that she had low bone mineral density (BMD) on DXA and was also provided with the diagnosis of Osteogenesis imperfecta few years back.

We advised for a complete metabolic workup. Her biochemical investigation showed serum calcium 9.1 mg/dl (while on calcium supplement), serum phosphorus 2.2 mg/dl, serum 25OHD 27 ng/dl,1,25(OH)D 54pg/dl, iPTH 176pg/dl. 24 hours urinary chemistry showed the following: protein 78.4 mg/dl, phosphorus 275.5, potassium 18.26, sodium 69.49, creatinine 0.34mmol/24hrs and creatinine clearance 73.7ml/min. The patient was identified to have low phosphorus and high PTH with optimal vitamin D levels. A preliminary diagnosis of hypophosphatemic osteomalacia was made and genetic consult was sought. Sample for genetic testing of the following hypophosphatemic gene panel ALPL, CLCNS, CYP2787, CYP2R1, DMPI, ENPPI, FAH, FAM20C, FGF23, FGFR1, PHEX, SLC34A3, VDR was sent to Invitae Medical Genetic Testing Expert Trust in Sanfransisco California. A pathogenic variant c.871C > T ((p. Arg291) was identified on PHEX gene confirming the diagnosis of X linked hypophosphatemic rickets.

She was started with potassium phosphate in divided doses and alfacalcidiol once daily. She has been counseled for XLH in detail about the disease and its inheritance, she was provided with instructions to avoid fall. On follow up after four weeks time, her pain greatly improved and she was advised to repeat serum calcium, serum phosphate and plasma PTH.

## 2 Discussion

The diagnosis of many rare bone diseases has been limited by physician access to the most up-to-date information about availability of diagnostic tests and diagnostic criteria for these rare diseases. A variety of factors including lack of knowledge and/or experience, lack of resources, or failure to follow recommendations may interfere with a physician's use of effective diagnostic techniques, preventive measures, and timely managements. Although genetic tests are crucial to the diagnosis of many rare bone diseases [5] the ordering of a specific test or set of tests typically depends on a clinician's evaluation and ability to recognize clues pointing to conditions for which genetic testing may be warranted. However, availability of genetic testing in Pakistan is only available at few places, but the initial stages of diagnosis still depend on classic clinical practices including patient history, physical examination, use of laboratory investigations and application of clinical knowledge and reasoning skills. One dilemma for clinicians and patients is that many rare diseases have symptoms that accompany a number of common conditions and laboratory results may or may not be definitive. Physicians may consider common conditions that are consistent with the available information before considering rare conditions.

Rickets and osteomalacia represents a group of diseases of divergent causes, characterized by a defect in matrix mineralization [6]. The cause is any condition resulting in inadequate calcium or phosphate mineralization of bone osteoid resulting in either hypocalcemia or hypophosphatemia. Phosphate being sufficiently abundant in natural foods dietary phosphate deficiency is unlikely to develop except under conditions of extreme starvation. Phosphate homeostasis and plasma phosphate concentration depends primarily on the renal tubular transport of phosphate and disorders of renal phosphate wasting are the most common hereditary forms of rickets and osteomalacia in western countries [7]. ^R^enal hypophosphatemia can be calculated by measuring the tubular maximum for phosphate reabsorption (TMP/GFR) by routine biochemical investigations[8]. Reduced TMP/GFR with low serum phosphorus points is suggestive of renal phosphate wasting. In addition low or normal alkaline phosphatase, normal parathyroid levels and normal or reduced calcium levels would further support hypophosphatemic rickets/osteomalacia. Table 1 shows the types of hypophosphatemic rickets/osteomalacia encountered in clinical practice with distinct pathophysiological basis, gene affected and different approaches to treatment with subtle difference in the biochemical diagnosis by routine chemistries. Hence, genetic testing plays an important role in differentiating amongst the inherited hypophosphatemic conditions. With increase availability of therapeutic options; it is important to differentiate between the various hypophosphatemic conditions [9]. For example ADHR/O has similar manifestations, with clinical rickets, and inappropriately low levels of 1,25 (OH)_2_ D. The cause is mutations of FGF23 gene that result in resistance to degradation, and accumulation of high circulating levels of FGF23, and subsequent phosphaturia. The spectrum of disease in ADHR/O includes not only the classic presentation with early onset of hypophosphatemia and rickets, but also includes delayed onset of clinically evident disease [9]. in which patients present after puberty with hypophosphatemia at an age ranging from 14.5 to 45 years with complaints of bone pains, weakness and fatigue without having histories of rickets or lower extremity deformities as a child. Econs in 1997 suggested that apparently individuals who presented as adults were able to compensate for genetic defects initially but later lost the ability to compensate [10]. Treatment involves correction of abnormalities caand administration of calcitriol and phosphate. However, there is limited data on treatment of ADHR/O compare to XLH. Similarly, in HHRH, the accurate diagnosis has important therapeutic implications. Unlike XLH & ADHR/O, phosphate supplementation alone can bring in remission wheras addition of vitamin D can create complications such as hypercalcemia, nephrocalcinosis and renal damage.

**Table 1:** CLINICAL biochemical, molecular and treatment in different hypophosphatemic rickets/osteomalacia.

	Investigations	X-Linked hypophosphatemic osteomalacia (XLH)	Autosomal hypophosphatemic osteomalacia (ADHO)	Vitamin d resistent hypophosphatemic osteomalacia	Mc Cune albright syndrome (MAS)	Fibrodysplasia ossificans progressiva (FOP)
BLOODS	S. CALCIUM	Normal	Normal	Decreased	Normal/Increased	Normal
	S. PHOSPHATE	Decreased	Decreased	Decreased	Decreased	Decreased
	Alkaline phosphatase	Increased/Normal	Increased	Increased	Increased	Increased
	25 OHD	Normal	Normal	Normal	Normal	Normal
	1,25 (OH)D	Normal/Decreased	Normal/Decreased	Normal	Normal	Normal
	PTH	Normal/Increased	Normal/Increased	Increased	Increased	Increased
URINE	URINARY CALCIUM	Normal	Normal	Increased	Normal	Normal
	URINARY PHOSPHATE	Increased	Increased	Increased	Increased	Increased
	TMP/GFR	Decreased	Decreased	Decreased	Decreased	Decreased
CLINICAL PRESENTATION	AGE OF PRESENTATION	7–8 years	5–7 years 14.5–45 years	6–7 years	15 -20 YARS	13–20s
	BONE DEFORMITIES	Short stature, bowing of legs, Multiple fracture history, cranial bossing	Bone pain Fatigue Muscle weakness fractures	Leg bone deformities, Cranial deformities, frontal bossing.	Not apparent bony deformities. CAFÉ-‘aulet spots, Mosaic like skin pigmentation Endocrinopathies.	Bone pain, Heterotopic ossification of soft tissues, immobility, swelling of femur, tibia, ribs and skull
	DENTAL DEFORMITIES	Dental carries, Tooth deformities	Tooth access	Delayed tooth eruption	Swelling of the jaws. Fibrous lesion of the jaws	
MOLECULAR PATHWAYS		PHEX Mutation on 22.2 and 22.1 on X chromosome	Heterozygous Point mutation on amino acid 176 or 179 at FGF23	Genetic defect in conversion of 25 hydroxyvitaminD2 to 1,25 dihydroxy vitamin D and increased levels of FGF 23.	Embryonic postzygotic activation in GNAS 1 gene encoding cAMP pathways associated G protein and Gas.	Mutation on ACVR1/ALK2 gene on chromosome 2q 24
MAIN MODE OF TREATMENT		Vitamin D supplements, oral phosphate supplements, Burosumab	Oral Phosphate supplements and calcitriol	Oral phosphate and calcitriol supplements	Bisphosphonates, Strengthening exercises, Surgical interventions.	Glucocorticoids to avoid the infections, surgical intervention

XLH is rare but commonest among other inherited forms of rickets. Most commonly occurs in children (1 in 20,000) but often misdiagnosed in childhood and may manifest as osteomalacia in adults [7]. The pathophysiology involves the loss of phosphate regulating gene with the homology to endopeptidase located on the X chromosome (PHEX) [11,12]. PHEX enzymes are involved in phosphate homeostasis through regulation of a protein called Fibroblasr growth factor (FGF23), secretd by bone osteoclasts and osteoblasts [11]. FGF 23 activates the renal KLOTHO receptors 1 that inhibit the phosphate reabsorption by downregulation of Napi2a and Napi2c expressions and sodium-phosphate co-transporter in the proximal renal tubules resulting in hypophosphatemia. FGF23 can reduce the regulation of CYP271B (that encodes 25OHD-1@-Hydroxylase) and upgrade the control of CYP24A1 (that encode 24-Hydroxylase) [13]. As a result there is lower level of 1,25OH_2_D due to decreased synthesis and increased catabolism. FGF23 is involved in several phosphate wasting disorders including Autosomal dominant hypophosphatemic rickets/osteomalacia (ADHR/O), oncogenic hypophosphatemic osteomalacia (OHO), McCune-Albright syndrome (MAS), fibrous dysplasia of bone (FD), and humoral hypercalemia of malignancy [9].

In Pakistan the first case of hypophosphatemic rickets was reported in 2004 diagnosis were made on the basis of family history and lab investigations. Now we report the second case of XLH, confirmed on genetic testing.This case report provides a new doorway to the investigations for physicians/clinicians. It is a possibility that there is underreporting of such cases. In the past this patient was given the diagnosis of osteogenesis imperfecta which is associated with type 1 collagen deficiency. While fragility fractures are common in both, the biochemical investigations are within normal limits in OI in contrast to XLH. Hence, it is important to screen with appropriate lab investigation. Patient was councelled regarding the XLRH and prescribed with calcium phospahtes and vitamin D supplements.

One relatively newer drug option is that of Cinacalcet; which is a calcimimmatics and regulates the parathyroid hormone by decreasing the extracellular calcium and reducing the parathyroid levels[14]. Recently, Burosumab (Crysvita, Ultragenyx) has been approved by FDA for the treatment of hypophosphatemic rickets and recommended as the first line of treatment for the adults and children older then 1 year suffering from hypophosphatemic rickets. Burosumab is an antibody that binds the FGF23 in XLH patients normalizing the phosphorus levels and improving the bone mineralization[15].

International data shows that early diagnosis and improved treatment for a number of bone disorders have increased the number of children who survive to adulthood with improved quality of life. Genetic counseling is also important step for individuals and families following the diagnosis to help them better understand the disorder, consider their options, and plan for the future. Family members may be advised about their options to be tested. Lack of availability and access to genetic counsellors in Pakistan is one negelcted area. There is a need to educate and assist physicians about the rare bone diseases in Pakistan for improving the provision of care. Need for development of local clinical practice guidelines, continuing medical education activities, development of centers of excellence for rare skeletal disease and/or establishing referral centers is needed in a resource limite country like Pakistan. The centers of excellence can also provide a base for research and may act as nidus for establishment of a network of accredited care centers to promote multidisciplinary, multicenter, collaborative research and more resources for translational research. In addition, centers can bring together comprehensive expertise and resources to address patient needs, and to monitor the care provided to the patient suffering from rare skeletal disorders.

## Provenance and peer review

Not commissioned, externally peer-reviewed.

## Competing intrest/funding

None.

## Contribution

All authors participated equally and take full responsibility of manuscript.

## Annals of medicine and surgery

The following information is required for submission. Please note that failure to respond to these questions/statements will mean your submission will be returned. If you have nothing to declare in any of these categories then this should be stated.

### Please state any conflicts of interest

NO.

### Please state any sources of funding for your research

NO.

### Ethical approval

Not applicable.

### Consent

Written informed consent was obtained from the patient for publication of this case report. A copy of the written consent is available for review by the Editor-in-Chief of this journal on request.

### Author contribution

AHK: conceived the idea, followed the patient, wrote the manuscript, made the critical revisions and take the full responsibility of the manuscript.

NZ: Wrote the manuscript.

LJ: Made the critical review and revise the manuscript. SK: did the genetic consult and performed the critical review.

### Registration of research studies

1. Name of the registry: N/A
2. Unique Identifying number or registration ID: N/A
3. Hyperlink to your specific registration (must be publicly accessible and will be checked):

## Guarantor

Dr Aysha Habib Khan MD, FCPS. FRCP.

Professor of Chemical Pathology & Bone Consultant,

Department of Pathology & Laboratory Medicine

Aga Khan University, Karachi, Pakistan

+92 (21) 34930051

E-mail: aysha.habib@aku.edu.
